# SARS‐CoV‐2 and human milk: What is the evidence?

**DOI:** 10.1111/mcn.13032

**Published:** 2020-05-30

**Authors:** Kimberly A. Lackey, Ryan M. Pace, Janet E. Williams, Lars Bode, Sharon M. Donovan, Kirsi M. Järvinen, Antti E. Seppo, Daniel J. Raiten, Courtney L. Meehan, Mark A. McGuire, Michelle K. McGuire

**Affiliations:** ^1^ Margaret Ritchie School of Family and Consumer Sciences University of Idaho Moscow Idaho USA; ^2^ Department of Animal and Veterinary Sciences University of Idaho Moscow Idaho USA; ^3^ Department of Pediatrics and Larsson‐Rosenquist Foundation Mother‐Milk‐Infant Center of Research Excellence (MOMI CORE) University of California San Diego California USA; ^4^ Department of Food Science and Human Nutrition and Institute of Genomic Biology University of Illinois Urbana Illinois USA; ^5^ Department of Pediatrics, Division of Allergy and Immunology University of Rochester School of Medicine and Dentistry Rochester New York USA; ^6^ Eunice Kennedy Shriver National Institute of Child Health and Human Development (NICHD) National Institutes of Health (NIH) Bethesda Maryland USA; ^7^ Department of Anthropology Washington State University Pullman Washington USA

**Keywords:** breastfeeding, breast milk, coronavirus, COVID‐19, human milk, infectious disease, SARS‐CoV‐2

## Abstract

The novel coronavirus SARS‐CoV‐2 has emerged as one of the most compelling and concerning public health challenges of our time. To address the myriad issues generated by this pandemic, an interdisciplinary breadth of research, clinical and public health communities has rapidly engaged to collectively find answers and solutions. One area of active inquiry is understanding the mode(s) of SARS‐CoV‐2 transmission. Although respiratory droplets are a known mechanism of transmission, other mechanisms are likely. Of particular importance to global health is the possibility of vertical transmission from infected mothers to infants through breastfeeding or consumption of human milk. However, there is limited published literature related to vertical transmission of any human coronaviruses (including SARS‐CoV‐2) via human milk and/or breastfeeding. Results of the literature search reported here (finalized on 17 April 2020) revealed a single study providing some evidence of vertical transmission of human coronavirus 229E; a single study evaluating presence of SARS‐CoV in human milk (it was negative); and no published data on MERS‐CoV and human milk. We identified 13 studies reporting human milk tested for SARS‐CoV‐2; one study (a non‐peer‐reviewed preprint) detected the virus in one milk sample, and another study detected SARS‐CoV‐2 specific IgG in milk. Importantly, none of the studies on coronaviruses and human milk report validation of their collection and analytical methods for use in human milk. These reports are evaluated here, and their implications related to the possibility of vertical transmission of coronaviruses (in particular, SARS‐CoV‐2) during breastfeeding are discussed.

**Key messages**

- Very little is known about coronaviruses in human milk and whether breastfeeding is a possible mode of vertical transmission.
- Limited, weak evidence suggests that some coronaviruses (including SARS‐CoV‐2) may be present in human milk, but these studies do not report methods of sample collection and validation of reverse transcription polymerase chain reaction (RT‐PCR) assays for human milk.
- Nothing is known about the timing of the antibody response in human milk to SARS‐CoV‐2 infection.
- Future research should utilize validated methods and focus on both potential risks and protective effects of breastfeeding.

## 1 INTRODUCTION

The global pandemic caused by the SARS‐CoV‐2 virus is one of the most compelling and concerning global health crises of our time. Fortunately, this pandemic has rapidly mobilized the full range of expertise represented by researchers, clinicians and public health officials. Although our understanding of the biology, clinical implications and strategies for mitigation continues to evolve, one issue that has received limited attention is the implication of this pandemic for infant feeding practices. This lack of attention has resulted in mixed messages regarding guidance about optimal infant feeding practices (e.g., American Academy of Pediatrics, 2020; Centers for Disease Control and Prevention, 2020a; World Health Organization, 2020a; United Nations Children's Fund [UNICEF], 2020) and a consequent lack of confidence about best approaches to infant feeding in the face of this growing pandemic. Even when a mother is positive for COVID‐19, the World Health Organization (WHO) recommends breastfeeding be initiated within 1 h of birth, exclusive breastfeeding be continued for 6 months and breastfeeding be continued for up to 2 years. They suggest use of appropriate respiratory hygiene, hand hygiene and environmental cleaning precautions. The UNICEF recommends that COVID‐19‐positive mothers continue breastfeeding while applying precautions, such as wearing a mask and handwashing before and after feeding (UNICEF, 2020). The U.S. Centers for Disease Control and Prevention (CDC) neither recommends nor discourages breastfeeding but advises that decisions be made by the mother and family in consultation with their health care providers (Centers for Disease Control and Prevention, 2020a). They recommend that during temporary separation (should that occur), mothers who intend to breastfeed should express their milk using proper hand hygiene and that the expressed milk should be fed to the newborn by a healthy caregiver. Further, if a mother and newborn do room‐in and the mother wishes to feed at the breast, the CDC recommends that she should wear a facemask and practice hand hygiene before each feeding.

It is well established that viral transmission through human milk can occur (Jones, 2001; Lawrence & Lawrence, 2004). Notable examples include human immunodeficiency virus (HIV; Black, 1996; Ziegler, Johnson, Cooper, & Gold, 1985), cytomegalovirus (CMV; Stagno & Cloud, 1994) and human T‐cell lymphotropic virus type 1 (HTLV‐1; Boostani, Sadeghi, Sabouri, & Ghabeli‐Juibary, 2018). Perhaps the most prominent example of mother‐to‐child viral transmission via breastfeeding is HIV infection, during which higher milk and serum viral loads are associated with an increased risk of transmission (Davis et al., 2016; Semba et al., 1999; Willumsen et al., 2003). The risk of postnatal infection for breastfed infants of HIV+ mothers is ≈10–20% over the first 2 years of life without the use of antiretroviral therapies (ART; Dunn, Newell, Ades, & Peckham, 1992; Nduati et al., 2001). However, compared with mixed feeding, exclusive breastfeeding is associated with lower risk of transmission of HIV infection to infants (Coutsoudis et al., 2001; Iliff et al., 2005). In many high‐income nations, breastfeeding is contraindicated in the case of maternal HIV infection with or without maternal ART (e.g., American Academy of Pediatrics, 2012; Centers for Disease Control and Prevention, 2020b). Conversely, in low‐and‐middle‐income nations, infant mortality from malnutrition and infectious disease may outweigh the risk of acquiring HIV via vertical transmission during breastfeeding. As such, breastfeeding is recommended (World Health Organization, 2016).

With respect to CMV, it is estimated that 60–70% of breastfed infants of CMV‐seropositive mothers become infected with CMV (Dworsky, Yow, Stagno, Pass, & Alford, 1983; Minamishima et al., 1994). The risk of CMV infection in neonates is highest in preterm and very low birthweight (less than 1,500 g) infants (Hamprecht & Goelz, 2017; Lanzieri, Dollard, Josephson, Schmid, & Bialek, 2013). A small percentage of infected infants develop a severe complication known as CMV sepsis‐like syndrome, which can be fatal (Fischer et al., 2010). Nonetheless, breastfeeding is not contraindicated in CMV‐seropositive women with healthy, term infants (American Academy of Pediatrics, 2012; Centers for Disease Control and Prevention, 2019b; World Health Organization, 2009).

For HTLV‐1, breastfeeding is considered the major route of infection for infants (Moriuchi, Masuzaki, Doi, & Katamine, 2013). HTLV‐1 infection is lifelong, and although most infected individuals remain asymptomatic, approximately 10% develop severe disease, including adult T‐cell leukaemia, a highly aggressive and usually fatal malignancy (Rosadas & Taylor, 2019). Some organizations and agencies list maternal HTLV‐1 as a contraindication for breastfeeding (American Academy of Pediatrics, 2012; Centers for Disease Control and Prevention, 2019a), whereas others do not (World Health Organization, 2009).

Human coronaviruses are enveloped, positive‐sense, single‐stranded RNA viruses first described in 1965 (Tyrrell & Bynoe, 1965). There are seven identified strains known to infect humans. Four of the strains (alphacoronaviruses 229E, NL63 and OC43 and betacoronavirus HKU1) are ubiquitous in humans and cause the common cold. There is limited evidence that one of these (229E) may be vertically transmitted from mothers to infants, although the mechanism remains unclear (Gagneur et al., 2008). The presence of 229E in neonatal gastric samples suggests that one possible mechanism for infection is through human milk, although this study did not evaluate human milk specifically (Gagneur et al., 2008).

In light of the emergence of the novel coronavirus SARS‐CoV‐2, several issues related to human milk and coronavirus infection demand immediate attention, the first and foremost being whether or not the virus is present in human milk produced by infected or exposed women. Of particular interest in this context are (1) the potential role that breastfeeding could play in vertical transmission of SARS‐CoV‐2 from women to infants via human milk and (2) the potential protective effects of targeted antibodies and other immunoprotective components in human milk against COVID‐19. The goal of this review was to evaluate the published evidence regarding the presence of this and other human coronaviruses in human milk.

## 2 METHODS

We used a variety of databases to identify relevant literature published as of 17 April 2020, and the list of databases and search terms used can be found in Table 1. It is noteworthy that in addition to using standard scientific databases (e.g., PubMed), we also used a general Google search and a search of preprint servers to identify reports that had not yet been published in refereed journals (i.e., grey literature). Any research in which human milk was collected and tested for a human coronavirus was included in this review. See Addendum in the supporting information online.

**TABLE 1:** Search terms, databases and preprint servers used to identify existing literature reporting the possibility of vertical transmission of coronaviruses from mother to infant during breastfeeding as of 17 April 2020

Databases and preprint servers searched	General breastfeeding terms	SARS‐CoV‐2 and general coronavirus terms	SARS‐CoV terms	MERS‐CoV terms
Google Scholar	Milk	SARS‐CoV‐2	SARS‐CoV	MERS‐CoV
Medline	Human milk	Coronavirus	SARS	MERS
National Library of Medicine, PubMed	Breast	Novel coronavirus	SARS‐CoV‐1	
	Breastfeeding	Human coronavirus		
Web of Science	Breastmilk	COVID‐19		
Google	Lactation	COVID		
bioRxiv	Virus transmission			
medRxiv	Mother‐to‐child			
Research Square	Child‐to‐mother			
Preprints	Vertical			

### 2.1 Ethical considerations

This review does not constitute human subject research, and as such, ethical approval was not required.

## 3 RESULTS

### 3.1 MERS‐CoV

The deadliest of the human coronaviruses to date is MERS‐CoV, which emerged in Saudi Arabia in 2012. The disease caused by MERS‐CoV, Middle Eastern respiratory syndrome (MERS), is characterized by severe respiratory illness with symptoms of fever, cough and shortness of breath. MERS‐CoV is a betacoronavirus, and the case fatality rate of MERS is 34% (Mahase, 2020). There are no reports of the presence or absence of MERS‐CoV in human milk. However, there are reports of the presence of MERS‐CoV in the milk of dromedary camels (*Camelus dromedaries*; Conzade et al., 2018; Hemida et al., 2015; Reusken et al., 2014), and there is one report of a human likely infected through the consumption of raw (unpasteurized) camel milk (Memish et al., 2014). In camel milk samples spiked with MERS‐CoV, viable virus could still be recovered after 48 h (van Doremalen, Bushmaker, Karesh, & Munster, 2014). These observations resulted in recommendations against consuming raw, unpasteurized camel milk (World Health Organization, 2019). It is unclear if there is vertical transmission of MERS‐CoV between camelid cows and their calves, and whether infection occurs as a direct result of lactation/nursing in this species. There are no data on vertical transmission of MERS‐CoV between women and their infants (Jeong et al., 2017; Schwartz & Graham, 2020).

### 3.2 SARS‐CoV

A related virus, SARS‐CoV, emerged in 2003 in China, although the disease (severe acute respiratory syndrome [SARS]) quickly spread globally. SARS is clinically manifested by fever, dry cough, headache, muscle aches and difficulty breathing. No treatment exists except supportive care, but there have been no reports of SARS‐CoV transmission since 2004. Like MERS‐CoV, SARS‐CoV is a betacoronavirus, and the case fatality rate of SARS is estimated at 10% (Mahase, 2020).

Currently, there is one report in which human milk was tested for SARS‐CoV (Robertson et al., 2004) and two reports of human milk being tested for SARS‐CoV antibodies (Robertson et al., 2004; Stockman, Lowther, Coy, Saw, & Parashar, 2004). Robertson and colleagues described a woman infected during the second trimester of pregnancy (19 weeks). A single milk sample was collected 131 days after the onset of symptoms, but no additional detail on the collection methodologies was provided. Milk was submitted to the CDC, where it was analysed using reverse transcription polymerase chain reaction (RT‐PCR) for viral nucleic acids, and enzyme immunoassay and indirect immunofluorescence to evaluate antibody presence. No additional details on analytical methods were provided. Although no viral RNA was detected, antibodies to SARS‐CoV were identified in the milk. The infant in this study was never tested for SARS‐CoV infection. Stockman and colleagues described a 38‐year‐old woman infected in the first trimester of pregnancy (7 weeks). She recovered fully and delivered a healthy male infant at 36 weeks of gestation. Milk samples were collected at 12‐ and 30‐day post‐partum and tested for SARS‐CoV antibodies; all were negative. No details on the collection or analysis of the milk were provided. The infant in this study tested negative for SARS‐CoV. In both these studies, it is possible that the women had stopped shedding the virus before the milk samples were collected. SARS‐CoV shedding in other biological samples typically peaks 12–14 days after the onset of disease (Cheng et al., 2004). There are no documented cases of vertical transmission of SARS‐CoV between mothers and infants (Schwartz & Graham, 2020).

### 3.3 SARS‐CoV‐2

The novel coronavirus SARS‐CoV‐2 was named after SARS‐CoV due to its shared sequence homology (77.9%; Kim et al., 2020) and similar clinical characteristics. The first reported cases of SARS‐CoV‐2 infection emerged in late 2019 in China. Although the current estimated case fatality rate for COVID‐19 (the disease caused by the SARS‐CoV‐2 virus) is much lower than those of SARS and MERS at roughly 2% (Mahase, 2020), the spread of this pathogen has been much more rapid and extensive.

At the time of writing, there were 13 studies (seven case studies and six case series; three of which were preprints or preliminary reports that had not been formally peer‐reviewed; Table 2) reporting direct testing of milk produced by women infected with SARS‐CoV‐2 (Chen et al., 2020; Dong et al., 2020; Fan et al., 2020; Y. Li et al., 2020; Liu, Wang, Li, et al., 2020; Liu, Wang, Zhang, et al., 2020; Salvatori et al., 2020; Wang et al., 2020; Yu et al., 2020; Wu et al., 2020) or by women whose infants were infected (Cui et al., 2020; Kam et al., 2020; Yuehua et al., 2020). In total, 48 milk samples produced by 32 women had been tested; all but one sample (Wu et al., 2020; non‐peer‐reviewed preprint) were negative for the presence of the virus. Two milk samples produced by a single woman were tested for SARS‐CoV‐2 specific antibodies; IgG but not IgM was identified in both samples (Yu et al., 2020; non‐peer‐reviewed preprint). A description of the relevant characteristics for the women and infants in these studies can be found in Table 2. Investigators conducting nine of the 13 studies analysed milk samples collected at birth or shortly thereafter, reporting only findings in colostrum or transitional milk. Those same nine studies reported on the milk produced by women who were infected during the third trimester of pregnancy, whereas the other four reported findings from milk produced by mothers of infants infected at 1.5, 3, 6 and 13 months of age (Cui et al., 2020; Kam et al., 2020; Yu et al., 2020; Yuehua et al., 2020). For the infants born to women infected during pregnancy, most were immediately separated from their mothers post‐delivery and were not breastfed for the duration of the period observed in their respective reports. Fourteen of the 32 infants described in these reports were born via caesarean section; only two were specified as vaginal births. Repeated milk samples, collected up to 27 days apart, were analysed for eight of the women. All the studies were conducted in China (Chen et al., 2020; Cui et al., 2020; Dong et al., 2020; Fan et al., 2020; Y. Li et al., 2020; Liu, Wang, Li, et al., 2020; Liu, Wang, Zhang, et al., 2020; Wang et al., 2020; Yuehua et al., 2020, Yu et al., 2020; Wu et al., 2020), Singapore (Kam et al., 2020) or Italy (Salvatori et al., 2020).

**TABLE 2:** Characteristics of women and infants for whom human milk has been sampled and tested for SARS‐CoV‐2 using RT‐PCR and for SARS‐CoV‐2 specific antibodies

Publication	Peer reviewed	Subjects (*n*)	Location	Repeated samples	Time of milk collection	Maternal age (year)	Gestational age at time of maternal infection	RT‐PCR results	Milk antibody results	Infant age at the time of infant infection	Infant sex	Delivery mode	Infant breastfed
Chen et al. (2020)	Yes	6^a^	China	No	Day 1	27	38 weeks, 2 days^b^	−	NA	NA	NS	Caesarean	NS
						26	36 weeks, 2 days^b^	−		NA	NS	Caesarean	NS
						26	38 weeks, 1 day^b^	−		NA	NS	Caesarean	NS
						26	36 weeks, 3 days^b^	−		NA	NS	Caesarean	NS
						28	38 weeks^b^	−		NA	NS	Caesarean	NS
						34	39 weeks, 4 days^b^	−		NA	NS	Caesarean	NS
Cui et al. (2020)	Yes	1	China	Yes	55–57 days	NS	NA	−	NA	50 days	Female	NS	Yes
Dong et al. (2020)	Yes	1	China	No	6 days	29	34 weeks, 2 days	−	NA	NA	Female	Caesarean	No
Fan et al. (2020)	Yes	2	China	Yes	Days 1 and 17	34	37 weeks	−	NA	NA	Female	Caesarean	No
				No	Day 1	29	36 weeks	−		NA	Female	Caesarean	No
Kam et al. (2020)	Yes	1	Singapore	No	6 months	NS	NA	−	NA	6 months	Male	NS	Yes^c^
Li et al. (2020)	Yes	1	China	Yes	Days 1, 2 and 3	30	35 weeks	−	NA	NA	Male	Caesarean	NS
Salvatori et al. (2020)	No	2	Italy	No	Day 18	36	NA	−	NA	<18 days	Male	NS	Yes
					Day 10	26	NA	−		<10 days	Female	NS	Yes
Liu, Wang, Zhang, et al. (2020)	No	2^d^	China	Yes	Days 2, 3 and 12	34	40 weeks	−	NA	NA	Male	Caesarean	No
				No	Day 2	30	37 weeks	−		NA	Unclear	Vaginal	NS
Liu, Wang, Li, et al. (2020)	Yes	10^e^	China	No	NS	NS	NS	−	NA	NA	Mixed	NS	No
Wang et al. (2020)	Yes	1	China	No	36 h	34	40 weeks	−	NA	NA	Male	Caesarean	No
Wu et al. (2020)	No	3^f^	China	Yes	Days 1, 6 and 27	29	35 weeks, 4 days	−	NA	NA	NS	Caesarean	NS
					Days 1, 6 and 27	28	35 weeks, 5 days	−		NA	NS	Caesarean	NS
					Days 1, 3, 6 and 27	27	38 weeks, 2 days	+		NA	NS	Vaginal	NS
Yu, Xu, Li, Hu, and Li (2020)	No	1	China	Yes	Days 1, 8, 15, 18 and 24^g^	32	NA	−^h^	IgG+, IgM−^i^	13 months	Male	NS	Yes
Yuehua et al. (2020)	Yes	1	China	No	3 months	NS	NA	−	NA	3.5 months	Female	NS	Yes

Abbreviations: NS, not specified; NA, not applicable; −, negative result; +, positive result.

^a^ Study presented data from nine women but only presented data related to milk produced by six women.

^b^ Gestational age upon admission.

^c^ The infant's breastfeeding status was not specified in the report, but it is presumed that he was at least partially breastfed as the mother was producing milk at 6‐month post‐partum.

^d^ Study presented data from three women but only presented data on the milk produced by two women.

^e^ Study presented data from 19 women but only presented data on the milk produced by 10 women.

^f^ Study presented data from 13 women but only presented data on the milk produced by three women.

^g^ Days are reported from time of admission, not from time of birth as the other days in this column.

^h^ Milk was analysed on Days 1, 8, 15 and 18 of hospital admission.

^i^ Milk antibodies were tested on Days 8 and 24 of hospital admission.

Wang et al. (2020) described a healthy, 34‐year‐old woman who acquired the infection in Week 40 of pregnancy. She gave birth to a male infant via caesarean section. The infant and his mother both tested positive for SARS‐CoV‐2 using pharyngeal swabs within 36 h of the delivery. The infant was separated from his mother at delivery and fed infant formula for the duration of the period described in the study. The mother's milk was collected at 36‐h post‐partum; it tested negative for SARS‐CoV‐2 via RT‐PCR. No description of the collection or testing methods was provided. The authors stated that they recommended the mother not breastfeed but instead pump milk to avoid mastitis.

In another case series from China, Fan et al. (2020) described two women who became infected during the third trimester of pregnancy. Patient 1 was 34 years old and in Week 37 of gestation at the time of diagnosis via RT‐PCR analysis of a nasopharyngeal swab. She delivered a female infant via caesarean section 6 days after testing positive for SARS‐CoV‐2 via nasopharyngeal swab. The infant was separated from the mother immediately after delivery, and serial tests of the infant's nasopharyngeal swabs were negative. A milk sample was collected within 24 h of delivery and 16 days later; both were negative for SARS‐CoV‐2. Patient 2 was 29 years old and in Week 36 of gestation at the time of diagnosis via RT‐PCR analysis of a nasopharyngeal swab. Her infant was delivered 5 days after she was diagnosed. A single milk sample was collected within 24 h of delivery; it tested negative for SARS‐CoV‐2. The authors of this report did not specify how the sample was collected, other than ‘breastmilk was obtained after the first lactation’.

Chen et al. (2020) described milk produced by six women infected during pregnancy. The women were 26–34 years old and between 36 weeks 2 days and 39 weeks 4 days of gestation at diagnosis. The authors did not provide details on the methods used for milk collection other than ‘breastmilk samples from patients with COVID‐19 pneumonia were collected after their first lactation’ and that milk was collected following WHO guidelines, but they did not provide a citation for this collection method. All milk tested negative for the virus, but no information was provided on the methods used for analysis.

In a report by Liu, Wang, Zhang, et al. (2020), milk produced by two women was tested. One woman was 34 years old and at 40‐week gestation tested positive for COVID‐19 via oropharyngeal swab. Milk was collected and tested at Days 1, 2 and 12 post‐partum; all samples were negative. Her male infant was delivered via caesarean and tested for SARS‐CoV‐2 via oropharyngeal swab when he was 1 and 7 days old; both swabs were negative. The other woman was 30 years old and delivered an infant vaginally after testing positive for SARS‐CoV‐2. Her infant tested negative at birth using an oropharyngeal swab; milk was collected on Day 2 post‐partum, and it was also negative. No details were provided for methods of collection or analysis.

In a case series by Liu, Wang, Li, et al. (2020), milk produced by 10 women infected during late pregnancy was tested via RT‐PCR for SARS‐CoV‐2; all samples tested negative. It is noteworthy that this report included data from 19 women, but milk was collected from only 10 of them. The authors did not specify for which of the women milk was collected. None of the 19 infants reported in this study tested positive for SARS‐CoV‐2 via RT‐PCR. The only detail available on collection or testing methods is that RT‐PCR was used to test the samples and that ‘milk was collected after the first lactation’. Despite their results, the authors concluded that delivery should occur in an isolation room and that infants be separated from infected mothers.

Li et al. (2020) described a 30‐year‐old woman at 35‐week gestation who was positive for SARS‐CoV‐2 and who delivered a male infant via emergency caesarean section. The infant was tested immediately upon delivery via oropharyngeal swab, which was negative. After delivery, the infant was kept in isolation away from his mother. Milk was collected immediately after delivery and on Days 2 and 3 post‐partum; all samples were negative. Again, no information on the collection or testing methods for the milk sample is available in this report.

Dong et al. (2020) described a 29‐year‐old woman at 34 weeks of gestation diagnosed with COVID‐19 via nasopharyngeal swab. Nearly a month later, the woman delivered a female infant via caesarean section. The infant was immediately separated from the mother with no contact. The infant consistently tested negative for SARS‐CoV‐2 via nasopharyngeal swab over the first 12 days of life. However, a blood sample at 2 h of age was positive for IgG and IgM antibodies to SARS‐CoV‐2. A milk sample was collected from the mother at Day 6 post‐partum; it tested negative for SARS‐CoV‐2 but was not tested for antibodies. No information on the collection or testing methods for the milk sample is included in the report.

In another case study, Yu et al. (2020; non‐peer‐reviewed preprint) described a 32‐year‐old woman with a 13‐month‐old breastfed male infant. Both the woman and her infant developed symptoms 2 weeks after exposure to infected family members and tested positive for COVID‐19 2 days after hospital admission. The woman insisted that she remain with her infant during the hospital stay, and the infant continued to breastfeed. Milk samples were collected and analysed for SARS‐CoV‐2 on Days 1, 8, 15 and 18 after admission, and all tested negative. Milk samples collected on Days 8 and 24 were tested for SARS‐CoV‐2 specific antibodies. In both samples, the authors identified IgG but not IgM. No details on method of milk collection, SARS‐CoV‐2 testing or antibody testing are provided in this report.

Wu et al. (2020; non‐peer‐reviewed preprint) are the only researchers to date who have reported a positive result for SARS‐CoV‐2 in a human milk sample. All the three women (27, 28 and 29 years old) whom they studied were infected during the third trimester of pregnancy and delivered infants via caesarean. Milk was collected from each woman on Days 1, 6 and 27 post‐partum into sterile containers after cleaning the breast with iodine; milk samples were tested via RT‐PCR for SARS‐CoV‐2. Whereas most samples were negative for the virus, the sample collected on Day 1 from the 29‐year‐old patient was positive. Subsequent testing of milk from this same subject 2 days later was negative for the virus. No details on the analytical methods used were provided. The infants were also tested for SARS‐CoV‐2 via both throat and anal swabs when they were 1 and/or 3 days old; all were negative.

In the only report from Italy, Salvatori et al. (2020) report on two women who were infected in the early post‐partum period. Patient 1, 36 years old, was admitted to the hospital at 18‐day post‐partum. Both she and her infant tested positive for SARS‐CoV‐2 via nasopharyngeal swab, but the infant was asymptomatic. Patient 2, 26 years old, was admitted at 10‐day post‐partum. She and her infant also tested positive for SARS‐CoV‐2, but her infant was symptomatic with diarrhoea and poor appetite. Both infants in this study were breastfed. A milk sample was collected from each woman on the day of admission; both tested negative for SARS‐CoV‐2 via RT‐PCR. No details on the collection or analytical methods used are reported in this study.

While the previous reports focused on infected women, there are also three case studies focused on infected infants. In these studies, milk produced by the infants' mothers was tested for SARS‐CoV‐2. The youngest of these infants was reported by Cui et al. (2020). After being exposed to infected family members, the 55‐day‐old female was admitted to the hospital with symptoms of COVID‐19 and diagnosed based on clinical data and exposure history. The infant was ‘mixed fed’. Her mother's milk was collected on the first three consecutive days of her hospitalization; all were negative for SARS‐CoV‐2. No information on the collection or testing methods for the milk sample is included in this report. Yuehua et al. (2020) reported on a 3‐month‐old, breastfed female who was hospitalized and tested via throat swab for SARS‐CoV‐2; the swab was positive. A single milk sample was collected from the infant's mother; it tested negative. The authors provided no information on the collection or testing methods for the milk. Importantly, this infant developed symptoms of COVID‐19 7 days before her parents became ill. As such, one possibility is that she was infected first and passed the infection to them. Another case report on a mature milk sample comes from Singapore (Kam et al., 2020). This report is particularly interesting as the infant had no symptoms but was hospitalized and tested because his caregivers were all hospitalized with COVID‐19, and there was no one to care for him. The infant was 6 months old and presumably at least partially human milk fed as a sample of milk was successfully collected from his mother. Despite being asymptomatic, a nasopharyngeal swab taken from the infant was positive for SARS‐CoV‐2. The authors reported that milk produced by the mother on a single day tested negative for the virus but did not specify how many samples were taken. This report provided no data on the methods used for the collection and analysis of these sample(s).

## 4 DISCUSSION

Despite the devastating clinical manifestations of MERS‐CoV, SARS‐CoV and SARS‐CoV‐2, there remains much to be learned about their modes of transmission. Respiratory droplets are a documented source of the virus (World Health Organization, 2020b), but other sources such as breastfeeding and/or human milk may exist. The primary purpose of this review was to examine the evidence (or lack, thereof) for the vertical transmission of SARS‐CoV‐2 from mother to infant via breastfeeding considering what is known about other human coronaviruses. We also examined the evidence presented in the same reports related to maternal/infant antibody production to the virus.

In total, we identified 14 studies that had tested human milk for human coronaviruses directly. Thirteen of these studies were newly published reports on SARS‐CoV‐2 and human milk, which collectively encompassed 48 milk samples. All but one of these samples tested negative for SARS‐CoV‐2, and that result was reported in a non‐peer‐reviewed, online preprint. We identified no comparable data for MERS; a single case report for SARS, which yielded a negative result for the presence of the virus but positive results for antibodies specific to SARS‐CoV; and no reports of human milk tested for other human coronaviruses. There was one report of antibody tests in milk specific to SARS‐CoV‐2, which identified IgG but not IgM (Yu et al., 2020).

The single milk sample that was supposedly positive for SARS‐CoV‐2 (Wu et al., 2020) is clearly anomalous to the larger body of evidence. Importantly, since no methodologies were provided and the study has not been published in a refereed journal, any interpretation of this result must be made with extreme caution. This underscores the fact that it is critical for the research community to focus its efforts on analysing appropriately collected human milk using laboratory methods that have been validated and optimized for the human milk matrix. In addition, results should be submitted to high‐quality, refereed journals for peer review. Only then can methods and results be rigorously assessed by scientists with the knowledge base needed to judge them. The dearth of high‐quality evidence substantially compromises the ability to effectively respond to this pandemic and provide guidance to some of the most vulnerable individuals: pregnant and lactating women and infants.

Limited and weak data suggest MERS may be present in camel milk, but the relevance to SARS‐CoV‐2 in human milk is unclear. Notably, Reusken et al. (2014) reported that milk analysed in the camelid studies was not collected aseptically; rather, samples were obtained according to local milking customs. As such, it is possible that the presence of MERS‐CoV in camel milk could be due to contamination from the milker, the calf or the environment, rather than milk representing an endogenous source of the virus. This is likely an issue with all the studies on SARS‐CoV‐2, where only one reported cleaning of the breast prior to sample collection (Wu et al., 2020). However, the limited data available on all three of these viruses (and human coronaviruses, in general) leave many questions unanswered with respect to the role, if any, of human milk in vertical transmission of coronaviruses.

One possible reason that most of the RT‐PCR results for the milk samples tested were negative is that the methods used were neither designed nor validated for human milk. Milk is a complex matrix containing substantial fat, DNases (Babina, Kanyshkova, Buneva, & Nevinsky, 2004), RNases (Das, Padhy, Koshy, Sirsat, & Rich, 1976; McCormick, Larson, & Rich, 1974; Ramaswamy, Swamy, & Das, 1993) and other PCR inhibitors (Abu Al‐Soud, Jonsson, & Radstrom, 2000; Al‐Soud & Radstrom, 2001; Schrader, Schielke, Ellerbroek, & Johne, 2012). Of note is the fact that commonly used silica column‐based RNA isolation methods are designed for a limited sample volume, and as such are not suitable for more voluminous liquid samples. Thus, validation of methods using human milk is needed (see Box 1). In addition, other than general statements about the timing of collection (e.g., ‘milk was collected after the first lactation’) and brief descriptions of the RT‐PCR assays used for nasal and throat swabs, none of the studies to date has described the methods of collection or how the milk was handled and stored in any detail. In addition, nothing is known about stability of SARS‐CoV‐2, if present, in human milk and how quickly (or at what temperature) it must be frozen to preserve fidelity. Information on sample collection, handling and storage is critical to evaluating whether the negative results described in these studies could be due to inadequate methods used.

**Box 1. Key points of assay validation**

Some factors to consider when validating methods for human milk testing of coronaviruses.

- Method of milk collection: use of manual milk expression versus electric pump, cleaning procedures of breast and pump, partial versus full breast expression and foremilk versus hindmilk.
- Sample handling and storage: container material, temperature and duration of refrigeration/freezing.
- Assay validation: nucleic acid extraction protocols, amplification protocols, reagent selection, proper positive and negative controls and fresh versus frozen milk.
- Viral quantification and viability: infectious dose and biologically relevant concentrations.

Another possibility is that there is low abundance of the virus in human milk, and it is often not captured in the limited samples tested so far. For example, in the report on other human coronaviruses by Gagneur et al. (2008), 159 maternal–infant dyads were tested (including 161 infants, two sets of twins). In this report, 229E was present in both maternal and infant samples in only two dyads. Additionally, in the milk of dromedary camels, MERS‐CoV appears to be present at very low abundance (Reusken et al., 2014). This suggests the possibility that a very low viral load in milk might also lead to an inflation of false negatives. Limited evidence from two patients suggests that SARS‐CoV‐2 shedding in respiratory samples peaks at ~6 days after onset of symptoms (Pan, Zhang, Yang, Poon, & Wang, 2020), indicating that timing of sample collection also plays an important role in virus detection.

Only one study has investigated antibodies in milk specific to SARS‐CoV‐2 (Yu et al., 2020; non‐peer‐reviewed preprint), and these researchers identified IgG in one milk sample produced by a woman at 13‐month post‐partum. Although limited to a single study, this finding combined with a large body of literature documenting targeted antibodies in human milk indicates that there may be a protective effect of breastfeeding when the mother is COVID‐19 positive. The infant in this study was older than all the other infants described here, was likely not exclusively breastfed (based on reported age) and likely had a more mature immune system than the youngest infants described in other reports. Still, further investigation into this finding is a critical next step in understanding how breastfeeding and/or the infant's consumption of the complex milieu of human milk impact the infant's immune response to and clinical manifestations of SARS‐CoV‐2 infection. This information is an important component in the risk/benefit analysis of developing evidence‐based breastfeeding recommendations related to maternal coronavirus infection.

Considering the observations by Yu et al. (2020; non‐peer‐reviewed preprint), other immune protective components of human milk should also be more thoroughly evaluated. Although the methods used to test this milk were not fully described, this observation could have impacts on the clinical management of infants born to women diagnosed with COVID‐19 during pregnancy and/or lactation. This observation is also supported by the findings of Dong et al. (2020) and Zeng et al. (2020) who reported that both IgG and IgM antibodies to SARS‐CoV‐2 were present in the serum of an infant within 2 h of age, despite multiple negative RT‐PCR tests of nasopharyngeal swabs over the first days of life. The presence of circulating antibodies at such an early stage of life could indicate transfer of SARS‐CoV‐2‐specific antibodies from mother to infant during gestation. However, it is noteworthy that IgM antibodies present in the serum of SARS‐CoV‐2 negative infants are not likely to have originated from the mother during gestation as IgM cannot cross the placental barrier due to size. From the limited data on SARS‐CoV, it appears that the presence of antibodies in milk could be influenced by timing of infection, where antibodies to SARS‐CoV were detected only in milk produced by a woman who acquired the infection later in pregnancy (Robertson et al., 2004). Together, these observations suggest that infant infection may occur in utero but that the virus may simply be absent from the upper respiratory tract immediately after birth and therefore undetectable on pharyngeal swabs.

Very recent work has demonstrated that, like SARS‐CoV and human coronavirus NL63 (Hofmann et al., 2005), angiotensin‐converting enzyme 2 (ACE2) is one of the receptors used by SARS‐CoV‐2 to enter host cells (W. H. Li et al., 2003; Yan et al., 2020). ACE2 is expressed across many body sites and tissue types, including the oral cavity (e.g., tongue and oral mucosa) and in mammary tissue (Xu et al., 2020). If mammary epithelial cells express this receptor, then it follows that viable virus could exist in milk. If it does, then the introduction of virus‐containing human milk could represent a mechanism of entry for SARS‐CoV‐2 and COVID‐19 infection for infants.

Another observation worth considering is that, in at least one of the reports (Yuehua et al., 2020), the infant was infected and symptomatic 7 days prior to the infant's parents. This suggests the possibility that a ‘reverse’ vertical transmission from infant to mother could occur, a phenomenon that has been observed for other pathogens, such as HIV (Belitsky, 1989; Little et al., 2012) and Ebola virus (Sissoko et al., 2016). One possible mechanism for maternal infection in this case is through retrograde flow, where milk and saliva move back into the mammary gland from the infant's mouth during suckling (Ramsay, Kent, Owens, & Hartmann, 2004). Although this mechanism is speculative, it represents a possible route whereby an infant could theoretically transfer a pathogen it has encountered in the environment to the mother. It is also possible that maternal infection could occur through other mechanisms, such as infant respiratory droplets (World Health Organization, 2020a) or via faecal matter (Xu et al., 2020).

To date, all reports on SARS‐CoV‐2 and human milk have originated in Asia (China and Singapore) or Europe (Italy). Although this limited geography makes sense given the fact that the initial epicentre of this pandemic was in Asia and this was followed by a large outbreak throughout Italy, studies from other globally representative populations are needed to make definitive conclusions regarding the possible presence and/or role of SARS‐CoV‐2 in human milk. Additionally, the importance of a coordinated, international effort by scientists, clinicians and public health officials to elucidate answers to the many remaining questions related to SARS‐CoV‐2 and breastfeeding cannot be overemphasized.

## 5 CONCLUSIONS

Human milk is the gold standard for infant feeding. However, confidence as to its safety and best practices around breastfeeding during maternal COVID‐19 infection has been compromised by the lack of rigorous evidence as to whether SARS‐COV‐2 can be vertically transmitted in milk and/or during breastfeeding. As such, there exists an immediate need to rapidly generate rigorous evidence for the role (if any) of human milk and breastfeeding in vertical transmission of COVID‐19 from mothers to infants. To accomplish this, validation of analytical methods for the human milk matrix, viability testing and evaluation of other immune components in milk will all be critical to this effort, especially given the known protective effects of breastfeeding in other infant respiratory infections (Box 2; Chantry, Howard, & Auinger, 2006; Duijts, Jaddoe, Hofman, & Moll, 2010). Substantial interdisciplinary research on this topic is required and should be performed rigorously and rapidly to best inform policies regarding early feeding choices and clinical management of breastfeeding mothers infected with SARS‐CoV‐2 and their infants.

**Box 2. Future needs**

To understand the role of human milk and SARS‐CoV‐2 infection, the following points must be rapidly addressed.

- Optimization of human milk collection and storage protocols for SARS‐CoV‐2 research.
- Validation of assays for identification of SARS‐CoV‐2 RNA and SARS‐CoV‐2‐specific immune components in human milk.
- Multinational population studies documenting presence or absence of SARS‐CoV‐2 virus and immune factors (including antibodies) in milk produced by infected women, women with infected infants and women who have been exposed to SARS‐CoV‐2; if the virus is identified in milk, its viability must be verified.
- Multinational population studies documenting (or not documenting) risk of COVID‐19 infections in breastfed versus nonbreastfed infants whose mothers are COVID‐19 positive.
- Research delineating implications of skin‐to‐skin breastfeeding versus consumption of pumped human milk.

## CONFLICTS OF INTEREST

The authors declare that they have no conflicts of interest.

## CONTRIBUTIONS

KAL, RMP and JEW performed the literature search. KAL wrote the first draft. All authors read, contributed to and approved the final manuscript.

## Supporting information

Supporting Information S1
