# Supplementary material for: SARS‐CoV‐2 and human milk: What is the evidence?
Source: Matern Child Nutr. 2020 May 30;16(4):e13032. doi: 10.1111/mcn.13032 (PMC7300480; doi:10.1111/mcn.13032)
Supplement: Supplementary file 1 — Supporting Information S1 [file MCN-16-e13032-s001.docx]

**ADDENDUM**

The COVID-19 pandemic represents a rapidly changing situation. Even in the span of days since we submitted this manuscript for peer review, several additional studies on SARS-CoV-2 and human milk have been published, either in refereed journals or on preprint servers. Collectively, these new papers report data garnered from milk produced by women living in Germany (Groß et al., 2020), Italy (Buonsenso et al., 2020; De Socio et al., 2020), Sweden (Gidlöf, Savchenko, Brune, & Josefsson, 2020), Turkey (Kalafat et al., 2020), Jordan (AlZaghal et al., 2020), Korea (Han et al., 2020), and China (Yang Li et al., 2020; Youjiang Li et al., 2020; Xiong et al., 2020; Yin et al., 2020). Importantly, data reported in most of these newly published papers support the conclusions drawn by us in our review – that is, there is little evidence that milk produced by women diagnosed with COVID-19 contains SARS-CoV-2. Indeed, at the time we wrote our review, there was only a single paper suggesting such evidence (Wu et al., 2020). However, data presented in two papers (Groß et al., 2020, non-peer-reviewed preprint; Buonsenso et al., 2020) published since that time now provide additional evidence that SARS-CoV-2 RNA can be detected in milk produced by some infected women. Details regarding these two papers are provided here so that the reader can better understand how they might add to the literature.

As noted in our review, one of the major shortcomings of the literature on this topic is that few details are reported regarding sample collection and analysis. With respect to these shortcomings, it is noteworthy that Groß and colleagues (2020, non-peer-reviewed preprint) have published the most methodologically comprehensive report to date. The two women described in this manuscript were diagnosed with COVID-19 while breastfeeding, and shared a hospital room prior to the diagnosis of subject 1, who first reported COVID-19 symptoms on d 3 postpartum and was diagnosed with COVID-19 (via positive swab) the following day. Milk (*n* = 4 samples) collected from subject 1 on d 12-14 postpartum tested negative for SARS-CoV-2, despite the fact that this woman tested positive again on d 15 postpartum. Subject 2 began reporting symptoms on d 4 postpartum and tested positive two days later. Milk samples (*n* = 4) collected from subject 2 tested positive for presence of SARS-CoV-2 RNA on d 8 (*n* = 2 samples), 10, and 11 postpartum. Subsequent milk samples (*n* = 3) collected on d 12 and 23 postpartum tested negative for SARS-CoV-2. Interestingly, this woman’s infant started showing signs of COVID-19 on d 8 and tested positive for SARS-CoV-2 on d 9 and d19 after previously testing negative on d 6 of age; on d 23 of life the infant tested negative. The authors reported high concordance of RT-PCR results targeting the SARS-CoV-2-N and -ORF1b genes, with peak detection on 10 d postpartum (4 d after the onset of symptoms). Peak viral load in milk samples testing positive for the virus was estimated as 9.48 x 10^4^ to 1.32 x 10^5^ genome copies/mL of skim and whole milk, respectively. Although they did not give detailed descriptions of milk collection methods, the authors noted that “based on specimen handling, internal controls, and negative testing of (subject 1’s) milk, contamination can be excluded.”

Buonsenso and colleagues (2020) also report testing milk for SARS-CoV-2 produced by 2 women with COVID-19. All 10 milk samples collected from subject 1 during d 1-3 and d 11-14 postpartum tested negative for SARS-CoV-2. Subject 1 fed her infant at the breast after being discharged from the hospital. Her infant tested positive on d 15 of life after testing negative on d 1 and 3. However, the infant never developed any signs of disease. For subject 2, 3 of 5 milk samples collected between d 1-5 postpartum tested positive for SARS-CoV-2, while none of the samples (*n* = 5) collected between d 14 -17 tested positive. This woman’s infant never tested positive for SARS-CoV-2 and was fed expressed human milk in a bottle by the infant’s father. No methods related to the collection or analysis of this milk were provided in this report.

Collectively, at this time there are at least 25 studies which report testing of human milk for SARS-CoV-2. While the vast majority do not provide evidence of the virus in milk, there are 3 studies (2 of which came out after our review went to press) that do. Of these 3 studies, one has been published in a peer-reviewed journal (Buonsenso et al., 2020); one is in press (Wu et al., 2020); and the other is still a non-peer-reviewed preprint (Groß et al., 2020). Nonetheless, it is our opinion that these new data do not change the overarching conclusion of our original review, which is that there is insufficient high-quality research to make evidence-based conclusions as to 1) how frequently the virus is present in human milk (the percentage of SARS-CoV-2 positive women with positive milk samples), 2) under what circumstances the virus appears in human milk, and 3) whether the virus can be transmitted via breastfeeding and if so, whether transmission via breastfeeding can cause COVID-19 in breastfed infants. If anything, the continued need for rigorous research on this topic has become even more clear. The need to understand the role that human milk plays or does not play in infant infection is critical, and we would like to reinforce our call to action: there remains an urgent need to conduct well-designed, controlled studies using optimized milk collection protocols and assays validated for human milk in a variety of globally representative populations. Until the complex interaction among maternal infection, lactation, feeding choices, and infant exposure/infection is better understood, developing evidence-based guidelines for breastfeeding women will remain impossible.

**References**

AlZaghal, L. A., AlZaghal, N., Alomari, S. O., Obeidat, N., Obeidat, B., & Hayajneh, W. A. (2020). Multidisciplinary team management and cesarean delivery for a Jordanian woman infected with SARS-COV-2: A case report. *Case Reports in Women’s Health*, e00212. https://doi.org/10.1016/j.crwh.2020.e00212

Buonsenso, D., Costa, S., Sanguinetti, M., Cattani, P., Posteraro, B., Marchetti, S., … Valentini, P. (2020). Neonatal late onset infection with severe acute respiratory syndrome coronavirus 2. *American Journal of Perinatology*. https://doi.org/10.1055/s-0040-1710541

De Socio, G. V., Malincarne, L., Arena, S., Troiani, S., Benedetti, S., Camilloni, B., … Francisci, D. (2020). Delivery in asymptomatic Italian woman with SARS-CoV-2 infection. *Mediterranean Journal of Hematology and Infectious Diseases*, *12*(1), e2020033. https://doi.org/10.4084/mjhid.2020.033

Gidlöf, S., Savchenko, J., Brune, T., & Josefsson, H. (2020). COVID‐19 in pregnancy with comorbidities: more liberal testing strategy is needed. *Acta Obstetricia et Gynecologica Scandinavica*, aogs.13862. https://doi.org/10.1111/aogs.13862

Groß, R., Conzelmann, C., Müller, J., Stenger, S., Steinhart, K., Kirchhoff, F., & Münch, J. (2020). Detection of SARS-CoV-2 in human breast milk. *MedRxiv*. https://doi.org/https://doi.org/10.1101/2020.04.28.20075523

Han, M. S., Seong, M.-W., Heo, E. Y., Park, J. H., Kim, N., Shin, S., … Choi, E. H. (2020). Sequential analysis of viral load in a neonate and her mother infected with SARS-CoV-2. *Clinical Infectious Diseases*. https://doi.org/10.1093/cid/ciaa447

Kalafat, E., Yaprak, E., Cinar, G., Varli, B., Ozisik, S., Uzun, C., … Koc, A. (2020). Lung ultrasound and computed tomographic findings in pregnant woman with COVID-19. *Ultrasound in Obstetrics & Gynecology*. https://doi.org/10.1002/uog.22034

Li, Yang, Zhao, R., Zheng, S., Chen, X., Wang, J., Sheng, X., … Sheng, J. (2020). Lack of vertical transmission of severe acute respiratory syndrome coronavirus 2, China. *Emerging Infectious Diseases*, *26*(6). https://doi.org/10.3201/eid2606.200287

Li, Youjiang, Hu, Y., Yu, Y., Zhang, X., Li, B., Wu, J., … Xu, J. (2020). Positive result of Sars‐Cov‐2 in faeces and sputum from discharged patient with COVID‐19 in Yiwu, China. *Journal of Medical Virology*. https://doi.org/10.1002/jmv.25905

Wu, Y., Liu, C., Dong, L., Zhang, C., Chen, Y., Liu, J., … Huang, H. (2020). Coronavirus disease 2019 among pregnant Chinese women: case series data on the safety of vaginal birth and breastfeeding. *BJOG: An International Journal of Obstetrics and Gynaecology*. https://doi.org/10.1111/1471-0528.16276

Xiong, X., Wei, H., Zhang, Z., Chang, J., Ma, X., Gao, X., … Pang, Q. (2020). Vaginal delivery report of a healthy neonate born to a convalescent mother with COVID­19. *Journal of Medical Virology*. https://doi.org/10.1002/jmv.25857

Yin, M.-Z., Zhang, L., Deng, G.-T., Han, C.-F., Shen, M.-X., Sun, H.-Y., … Chen, X. (2020). Severe acute respiratory syndrome coronavirus 2 (SARS-CoV-2) infection during pregnancy in China: a retrospective cohort study. *MedRxiv*, 2020.04.07.20053744. https://doi.org/10.1101/2020.04.07.20053744
